# A thalamo-centric neural signature for restructuring negative self-beliefs

**DOI:** 10.1038/s41380-021-01402-9

**Published:** 2022-01-01

**Authors:** Trevor Steward, Po-Han Kung, Christopher G. Davey, Bradford A. Moffat, Rebecca K. Glarin, Alec J. Jamieson, Kim L. Felmingham, Ben J. Harrison

**Affiliations:** 1 Melbourne Neuropsychiatry Centre, Department of Psychiatry, The University of Melbourne, Parkville, VIC Australia; 2 Melbourne School of Psychological Sciences, University of Melbourne, Parkville, VIC Australia; 3 The Melbourne Brain Centre Imaging Unit, Department of Medicine and Radiology, The University of Melbourne, Parkville, VIC Australia

**Keywords:** Neuroscience, Psychology, Psychiatric disorders

## Abstract

Negative self-beliefs are a core feature of psychopathology. Despite this, we have a limited understanding of the brain mechanisms by which negative self-beliefs are cognitively restructured. Using a novel paradigm, we had participants use Socratic questioning techniques to restructure negative beliefs during ultra-high resolution 7-Tesla functional magnetic resonance imaging (UHF 7 T fMRI) scanning. Cognitive restructuring elicited prominent activation in a fronto-striato-thalamic circuit, including the mediodorsal thalamus (MD), a group of deep subcortical nuclei believed to synchronize and integrate prefrontal cortex activity, but which has seldom been directly examined with fMRI due to its small size. Increased activity was also identified in the medial prefrontal cortex (MPFC), a region consistently activated by internally focused mental processing, as well as in lateral prefrontal regions associated with regulating emotional reactivity. Using Dynamic Causal Modelling (DCM), evidence was found to support the MD as having a strong excitatory effect on the activity of regions within the broader network mediating cognitive restructuring. Moreover, the degree to which participants modulated MPFC-to-MD effective connectivity during cognitive restructuring predicted their individual tendency to engage in repetitive negative thinking. Our findings represent a major shift from a cortico-centric framework of cognition and provide important mechanistic insights into how the MD facilitates key processes in cognitive interventions for common psychiatric disorders. In addition to relaying integrative information across basal ganglia and the cortex, we propose a multifaceted role for the MD whose broad excitatory pathways act to increase synchrony between cortical regions to sustain complex mental representations, including the self.

## Introduction

Beliefs that are negatively biased, inaccurate, and rigid play a key role in etiology and maintenance of psychopathology [1]. Cognitive models of mood disorders posit that maladaptive self-beliefs—for example, believing that one is inherently flawed or unlovable—are central to triggering the emotional disturbances characteristic of these disorders [2]. Cognitive-behavioral therapy (CBT) and other evidence-based psychotherapeutic treatments are centered on identifying and restructuring maladaptive cognitions, often through Socratic questioning techniques [3, 4]. In the context of CBT, Socratic questioning asks a series of carefully sequenced questions to help an individual define problems, assist in the identification of maladaptive thoughts and beliefs, examine the meaning of events, and to assess the consequences of thought patterns or behaviors. Through the Socratic questioning and cognitive restructuring process, the individual learns to identify and re-evaluate their perspective towards internal thoughts, and modify processes which contribute to the maintenance of maladaptive conceptualizations [5]. The extent to which an individual’s self-beliefs are malleable has been found to uniquely predict CBT outcomes, as well as long-term reductions in disorder severity [6]. Despite the importance of cognitive restructuring in psychotherapeutic interventions, the neurobiological mechanisms underpinning these processes remain largely enigmatic.

Neuroimaging studies have identified a consistent set of brain regions that support the cognitive reappraisal of negative emotions elicited by provocative stimuli, typically visual images. This network comprises the dorsolateral prefrontal cortex (dlPFC), pre-supplementary motor area (preSMA), and the dorsal anterior cingulate cortex (dACC) [7–9], whose increased activity consistently accompanies the successful down-regulation of emotion, together with the modulation of activity in other brain regions, including the amygdala [10, 11]. Whether this network also supports the cognitive restructuring of negative self-beliefs remains unclear. In a recent study of patients with social anxiety disorder, a more distinct involvement of the ‘default mode network (DMN)’ was reported when patients reacted to versus accepted negative self-beliefs [12]. This finding is broadly consistent with other studies linking DMN activity, particularly the medial prefrontal cortex (MPFC), to negative self-appraisal processes including depressive rumination [13, 14]. Based on such findings, it has been hypothesized that this core MPFC-based ‘self-network’ dynamically interacts with lateral PFC ‘control’ regions and affective value-signaling regions, including the striatum [15, 16]. Within this framework, subcortical network hubs are likely to be pivotal for integrating self-representations across distributed cortical regions in support of higher-order cognitive processing [17, 18].

Of these subcortical regions, the mediodorsal thalamus (MD) may be especially important to higher-order cognition due to its distinguishing arrangement of dense direct and converging innervations from multiple PFC regions [19]. These features have led to the suggestion that the MD serves as a relay that rapidly enables crosstalk between widely distributed PFC regions—a major shift from the ‘cortico-centric’ view of cognition [20]. However, experimental evidence indicates a more complex role for the MD involving the regulation of plasticity within the PFC, as well as providing flexibility to PFC-mediated cognitive functions. For instance, lesions to the MD have been found to cause anterograde amnesia and impairments in updating representations of expected outcomes, while sparing the retrieval of learned cues and behavioral strategies [21, 22]. These findings suggest that updating self-beliefs and learning from new representations in working memory—fundamental aspects of cognitive restructuring—are dependent upon the coordinated activity of cortical networks with the MD [23].

Furthermore, complementing its close partnership with the PFC, the MD receives dense afferent projections from basal ganglia structures, including the striatum [24]. In a noteworthy recent study, multiple large-scale cortical networks, spanning the DMN and lateral PFC, were shown to converge on primary subcortical connectivity zones in the MD and caudate nucleus—suggesting a putative mechanism for the dynamic integration of cognitive networks supporting aforementioned self and control processes [25]. Taken together, the divergent and convergent nature of MD projections offers a plausible architecture for integrating multiple information streams, thereby enabling the elaboration and modulation of complex mental representations required for cognitive restructuring [26].

**Fig. 1:**
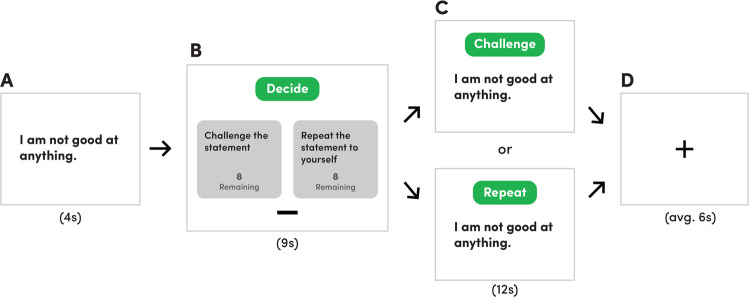
Cognitive restructuring paradigm. The task consisted of one run of 16 blocks. In each block, **A** a negative belief statement was presented for 4 s; **B** next, participants had 9 s to decide whether to challenge (CHAL) the statement using previously trained Socratic questioning techniques or to repeat (REP) the statement to themselves. During each block, participants were shown how many remaining choices they had for each option (CHAL or REP); **C** after their choice, participants either cognitively reframed or repeated the statement for 12 s; **D** a fixation cross was displayed for an average of 6 s before the next block began.

Our aim in the current study was to investigate the neural systems basis of restructuring negative self-beliefs with an emphasis on mapping integrative fronto-striato-thalamic circuit connectivity. To do so, we developed a novel regulation paradigm in which participants were trained to cognitively restructure negative self-beliefs using established Socratic questioning techniques (Fig. 1). To support the precise functional anatomical mapping of fronto-striato-thalamic regions, we utilized ultra-high field functional magnetic resonance imaging (fMRI), which we combined with dynamic causal modelling (DCM) to examine directed interactions (‘effective connectivity’) between identified task-responsive regions. Our principal hypothesis was that the cognitive restructuring of negative beliefs would elicit robust activation of a fronto-striato-thalamic network, encompassing the MD and caudate as major subcortical hubs, together with the MPFC, dACC/preSMA and dlPFC. Through DCM, we tested multiple architectures of fronto-striato-thalamic function with the specific goal of mapping the causal influence of the MD on broader network activity. Lastly, we examined whether participants’ tendency to engage in repetitive negative thinking—a trait construct broadly linked to affective disorders [1]—could be predicted by fronto-striato-thalamic interactions during cognitive restructuring.

## Materials and methods

### Participants

49 healthy adults were recruited for this study. Exclusion criteria included: (1) the presence of a serious mental illness (e.g., psychosis, bipolar disorder, obsessive-compulsive disorder) screened using the Mini-International Neuropsychiatric Interview (MINI; [27]; or (2) MRI contraindications (e.g., pregnancy, metallic implants or claustrophobia). We chose to examine the neural basis of restructuring negative self-beliefs in healthy samples in order to establish an initial foundation that could serve as a reference point for future investigations using clinical samples. Participants provided written informed consent and attended a single testing session at the Melbourne Brain Centre Imaging Unit (The University of Melbourne, Parkville). This study was approved by The University of Melbourne Human Research Ethics Committee.

Seven participants were excluded from this initial sample due to poor physiological recording quality (*n* = 3), not properly completing the task (*n* = 2), scanning being terminated early (*n* = 1), and excessive movement (*n* = 1). As a result, 42 healthy participants (age 24.7 ± 4.65 years) were included in the final DCM analysis. Supplementary Table S1 provides a summary of the sociodemographic characteristics of the final sample.

### Behavioral measures

Repetitive negative thinking was measured using the Perseverative Thinking Questionnaire (PTQ). The validity is of this questionnaire is supported by associations with existing repetitive negative thinking measures and by correlations with depression and anxiety symptom levels [28]. Internal consistency for the PTQ was excellent (Cronbach’s *α* = 0.908). All behavioral and connectivity data were checked for normality of distribution by Shapiro–Wilk tests before performing parametric statistical analyses.

### MRI paradigm

Further information on the MRI paradigm training procedures is available in the Supplementary Information.

The cognitive-restructuring task consisted of a single run containing sixteen blocks (see Fig. 1). In each block, participants were first presented with a negative self-belief statement for four seconds. Next, participants were given nine seconds to decide whether to challenge (i.e., cognitively reframe) or repeat the statement. Participants indicated their choices using an MRI-compatible control pad. To ensure an equal number of blocks for each condition, participants were instructed to only cognitively restructure half the statements and to repeat the remaining half of the statements. The remaining number of times that participants could challenge or repeat a statement was displayed alongside these two choices. After their choice, the same statement was displayed for twelve seconds, during which time participants engaged in their previously selected strategy. If the participant chose to restructure the negative belief, a prompt reading ‘Challenge’ was displayed alongside the statement, and participants were instructed to mentally refute or reinterpret the negative statement throughout the entire twelve seconds (herein referred to as ‘CHAL’). If the choice were to repeat, a prompt reading ‘Repeat’ was shown with the negative statement, and participants were instructed to mentally recite the negative belief until the twelve seconds had expired (herein referred to as ‘REP’). Between each statement block, a fixation cross was presented for an average of six seconds to reduce carry-over effects.

### Image acquisition

Information on 7-Tesla (7 T) image acquisition parameters, preprocessing and physiological noise correction (see Supplementary Fig. S1) is available in the Supplementary Information.

### First- and second-level General Linear Model (GLM) analyses

First-level (single-subject) contrast images were estimated for CHAL > REP (i.e., the entirety of the 12 s that ‘Challenge’ or ‘Repeat’ prompts were displayed) to characterize changes in brain activation associated with cognitive restructuring. Participant’s pre-processed timeseries and nuisance regressors (i.e., physiological noise and motion fingerprint regressors) were included in the GLM analysis, with the onset times for each condition event specified and convolved with the SPM canonical hemodynamic response function (HRF). A 128-Hz high-pass filter was applied to account for low-frequency noise. Temporal autocorrelation was estimated using SPM’s FAST method, which has been shown to outperform AR(1) at short TRs and yield superior reliability [29]. Contrast images for each participant were entered in a second-level random-effects GLM using a one-sample t-test design. For all GLM analyses, whole-brain, false discovery rate (FDR) corrected statistical thresholds were applied (P_FDR_ < 0.05), in addition to a 10-voxel cluster-extent threshold (K_E_ ≥ 10 voxels).

### Dynamic Causal Modelling (DCM)

DCM estimates the directional interactions between brain regions of interest through the process of generating timeseries from underlying neurobiological causes. These timeseries are dependent upon the connectivity architecture of the network and the strength of the connectivity parameters. Parameter strengths are estimated through model inversion, a process of finding the parameters that offer the best trade-off between model fit and model complexity. The relative evidence of these estimates can then be compared through model comparison, which tests hypothetical functional architectures to identify a model which optimally explains the data. In contrast to temporal correlation measures of functional connectivity, DCM provides a more detailed and physiologically valid mapping of effective connectivity—the directed causal influences of brain regions on one another [30]. In DCM, modulation is measured in hertz (Hz), which denotes the rates of change in activity caused by the dynamic influence of one region on another. Positive effective connectivity indicates a putative excitatory upregulation of activity, whereas negative connectivity represents an inhibitory downregulation by a task effect (i.e., cognitive restructuring). It has been shown that 7 T fMRI furnishes more efficient estimates of effective connectivity than those provided by lower field strengths [31].

In addition to the left MD, peaks from four regions displaying significant changes in activation during the CHAL > REP contrast were included in the model space: the left caudate, ventral MPFC, dlPFC and preSMA. The regional time-series (volumes-of-interest) for each of these areas were extracted at an individual subject level following recently published guidelines [32]. Further information on our volume-of-interest extraction procedure is included in the Supplementary Information.

**Fig. 2:**
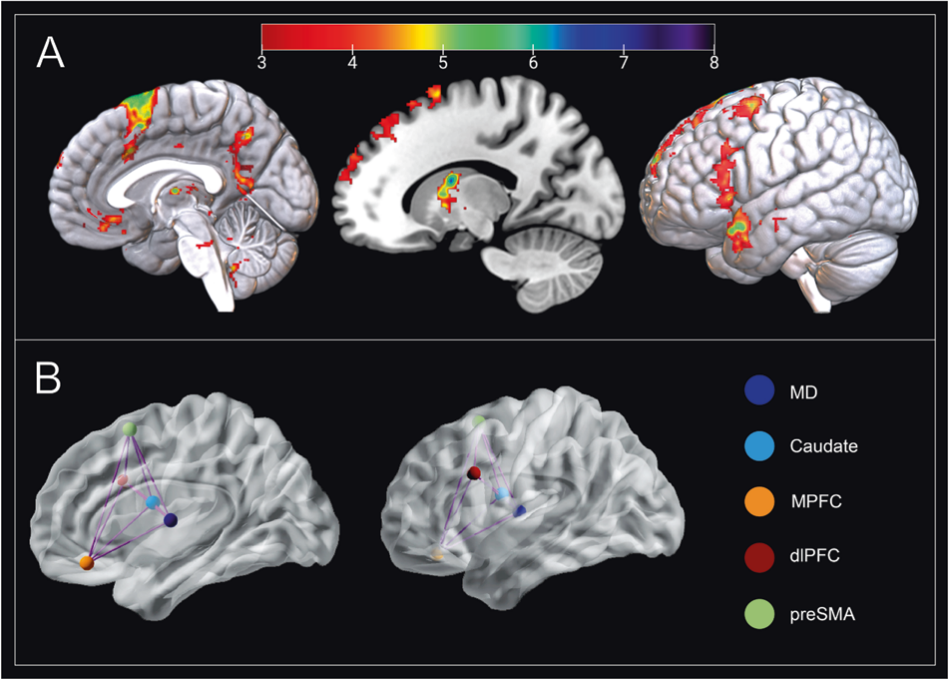
Neural response elicited by cognitive restructuring. **A** The mediodorsal thalamus (MD; *x* = −2; *y* = −10; *z* = 8), caudate (*x* = −16; *y* = 0; *z* = 18), medial prefrontal cortex (MPFC; *x* = −2; *y* = 37; *z* = −16), dorsolateral prefrontal cortex (dlPFC; *x* = −43; *y* = 16; *z* = 30), and pre-supplementary motor area (pre-SMA; *x* = −8; *y* = 11; *z* = 67) are responsive to cognitive restructuring (CHAL > REP). Group activation is overlaid on a MNI T1 template. Color bar represents *t*-values. **B** Our full model assumed bidirectional intrinsic connectivity between all five DCM nodes.

Our full model space was specified using DCM 12.5. Models varied by two components: the endogenous connections between nodes (intrinsic parameters) and the modulation of the strengths of functional coupling between the MD and other regions induced by cognitive restructuring. The full model assumed bidirectional endogenous connections between the MD with the caudate, ventral MPFC, dlPFC and the preSMA (Fig. 2B) and set ‘Task’, the onset of all blocks comprising both CHAL and REP blocks, as the driving input to all regions. In order to test the modulatory effect of cognitive restructuring on MD connectivity, CHAL was set as the modulatory input on each connection to and from the MD. Bayesian model reduction (BMR) tested whether modulation by CHAL occurred on the connections from the caudate, ventral MPFC, dlPFC, and preSMA to the MD, or from the MD to the caudate, ventral MPFC, dlPFC, and preSMA.

**Fig. 3:**
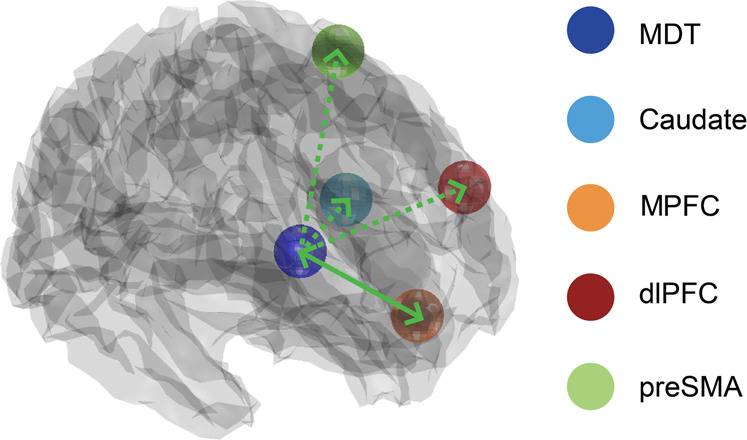
Mediodorsal thalamus effective connectivity and its relationship to cognitive restructuring. Carrying out cognitive restructuring led to excitatory effects on effective connectivity from the mediodorsal thalamus (MD) to the caudate, medial prefrontal cortex (MPFC), dorsolateral prefrontal cortex (dlPFC), and pre-supplementary motor area (pre-SMA; posterior probability > 0.95). In contrast, evidence was only found for excitatory effects from the ventral MPFC to the MD during cognitive restructuring (posterior probability > 0.95). Unilateral modulation of pathways from the MD is depicted with a broken green line. Bilateral modulations of MD pathways are depicted with a solid green.

Full models of effective connectivity were fitted to each participant’s timeseries data, yielding posterior connectivity parameters and their probabilities. At the group level, the posterior connectivity parameter estimates from all participants’ DCMs were assessed using Parametric Empirical Bayes (PEB) and BMR. The PEB framework affords robust group-level analyses of effective connectivity by means of a hierarchical model, comprising DCMs at the single-subject level and a GLM of connectivity parameters between subjects [33]. After estimating the PEB model, parameters that did not contribute to the model evidence were pruned using BMR. Posterior parameter estimates following BMR were averaged using Bayesian model averaging (BMA), and the ensuing BMA parameters (with a posterior probability >95%) are reported in the Supplementary information, Fig. S2. The resulting pattern of effective connectivity is illustrated in Fig. 3.

Last, we sought to test whether the effect of modulation of MD pathways by cognitive restructuring depended on individual participants’ tendency to engage in repetitive negative thinking. A PEB model containing a regressor quantifying the effects of PTQ scores on each of DCM modulatory parameter was examined, with parameters which did not contribute to model evidence being removed through pruning. Parameters featuring strong evidence (posterior probability >95%) were then tested using leave one-out cross validation to determine whether the size of these parameters were sufficiently large to predict a participant’s PTQ score. This procedure iteratively creates a PEB model on all expect for one subject, then predicts the PTQ score of the left-out subject. These complete results and further information on the modulatory effects of cognitive restructuring on the other regions in our model space are available in the Supplementary Information and at https://github.com/trevorcsteward/MD_Effective_Connectivity.

## Results

### Behavioral results

After extensive training on how to use Socratic questioning to challenge negative self-beliefs, participants completed our cognitive restructuring task during which they were presented with common negative self-belief statements during scanning (see Fig. 1 and Materials and Methods). We observed a significant decrease from pre- to post-scanning in participants’ endorsement of the negative self-beliefs statements that were restructured (50% of statements; *t*(48) = 7.85, *p* < 0.001), indicating that they successfully applied Socratic questioning to restructure negative self-beliefs. Participants were more likely to endorse stronger beliefs in statements that were repeated (*M* = 3.05 ± 1.20) compared to statements that were restructured (*M* = 2.57 ± 0.84; *t* = 4.39; *p* < 0.05), though the effect size was small (|d| = 0.421).

### GLM results

As hypothesized, whole-brain fMRI analysis (*p* < 0.05, voxelwise false discovery rate [FDR] corrected) confirmed that cognitive restructuring, compared to the task repeat condition, elicited significant activation of distributed frontal cortical and striatal-thalamic regions (Fig. 2A). Cortically, these regions included the MPFC, specifically its ventral-subgenual aspect; the preSMA extending to dACC, as well as the left dlPFC and frontal operculum. Subcortically, we observed prominent activation of the MD and head of the caudate nucleus, together with other basal ganglia regions including the globus pallidus and ventral putamen, as well as the midbrain periaqueductal gray. A complete anatomical description of these results is provided in Supplementary Table S2.

### DCM results

DCM utilizes a Bayesian framework to infer the causal architecture of a network of regions (i.e., nodes), defined in terms their ‘effective connectivity’—the extent to which a region’s activity directly influences another. Here, we used DCM to assess the modulatory impact of cognitive restructuring on MD effective connectivity with other key regions of interest, including the ventral MPFC, dlPFC, preSMA, and caudate (Fig. 2B). The full model of our network was designed to examine the modulatory effects of cognitive restructuring on pathways to- and from the MD. Following model estimation, BMR was used to iteratively test configurations of this neural architecture (i.e., the full model) and to prune any redundant parameters which did not contribute an increase in model evidence. In reduced models, the priors for a certain subset of connections may be switched off (i.e., fixed at zero). This approach is conducted under the assumption that all reduced models have equal priors a priori, and thus the ‘full’ model should only contain parameters that are biologically plausible. This method allows for the identification of regional connectivity parameters best explained by the data at a group level and for the verification of causal excitatory or inhibitory effects of MD circuits of brain activity during cognitive restructuring [34].

Consistent with previous work postulating an influence of the MD on cortical systems [19], BMR identified a strong excitatory effect of MD activity on the ventral MPFC, dlPFC, and preSMA during cognitive restructuring (posterior probability > 0.95; Fig. 3). MD activity also had an excitatory influence on the caudate (head) during cognitive restructuring—result that is consistent with evidence of direct anatomical connections between these regions [35]. Relevantly, the ventral MPFC was the only region identified to exert a reciprocal modulatory (excitatory) influence on MD activity, suggesting that this pathway may serve as the primary relay for PFC modulation of the MD across this network (Fig. 3 and Fig. S2). Additional results on the modulatory parameters between the caudate, preSMA, vmPFC, and dlPFC during cognitive restructuring are presented in Fig. S3.

### Mediodorsal thalamus modulation and repetitive negative thinking

**Fig. 4:**
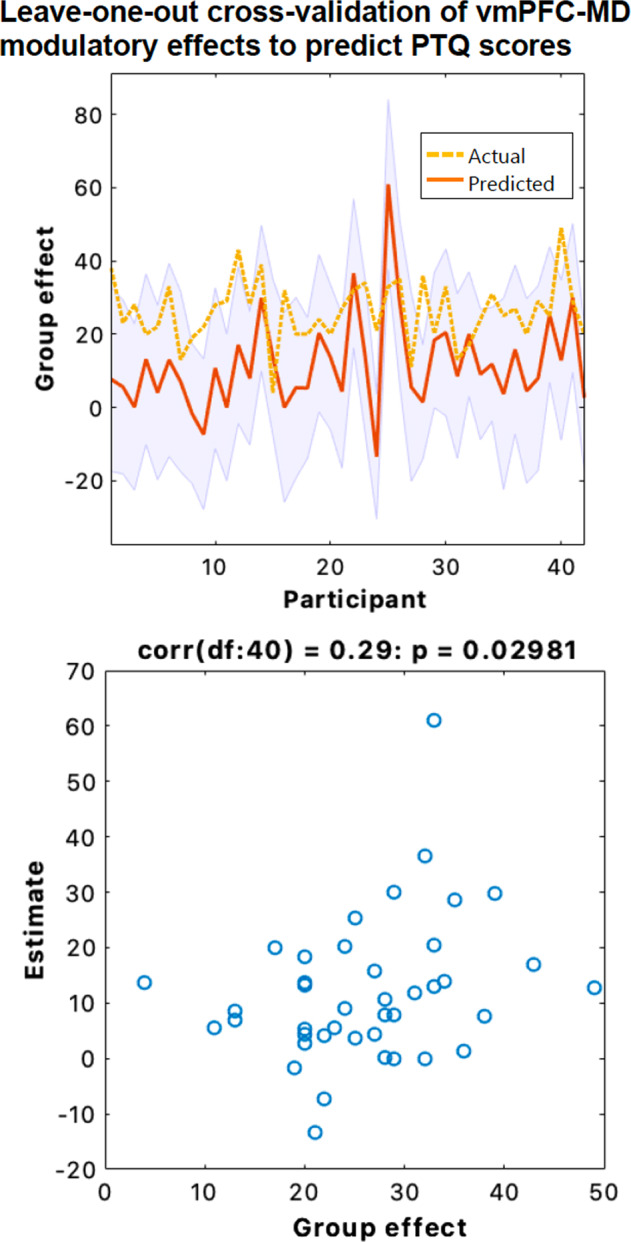
Leave-one-out cross validation of Parametric Empirical Bayes (PEB) effects on ventral MPFC-to-MD modulation during cognitive restructuring. Top panel: the out-of-samples estimate of Perseverative Thinking Questionnaire (PTQ) scores for each subject (red line) with 90% confidence interval (shaded area). The dashed orange line is the actual group effect. Bottom: The actual subject effect plotted against the expected value of the estimated subject effect. Participant PTQ scores can be reliably predicted based on their modulation of ventral MPFC-to-MD effective connectivity during cognitive restructuring (*p* = 0.029, *r* = 0.29).

Using estimates of connectivity strengths, we tested whether MD interactions during cognitive restructuring predicted participants’ tendency to engage in repetitive negative thinking. A Parametric Empirical Bayes (PEB) model was specified containing a parameter to quantify the effects of individual Perseverative Thinking Questionnaire (PTQ) scores on each MD pathway in our network. There was strong evidence to support an association between higher PTQ scores and greater excitatory influence of the ventral MPFC on MD activity during cognitive restructuring (posterior probability >0.95). Next, we used leave-one-out cross validation [34] to establish the predictive validity our model and found that individual ventral MPFC-to-MD connectivity strength levels could reliably classify participants’ PTQ scores (*p* = 0.029, *r* = 0.29; Fig. 4).

## Discussion

Our aim was to assess how the cognitive restructuring of negative self-beliefs is mediated by fronto-striato-thalamic pathways, with particular emphasis on the functionally integrative role of the mediodorsal thalamus (MD). As hypothesized, cognitive restructuring elicited prominent activation of DMN regions associated with self-directed thought, lateral PFC cognitive control regions, and subcortical hubs including the MD and head of the caudate. Using DCM, applied to ultra-high field fMRI data, we found strong evidence for the MD having an excitatory effect on the PFC and caudate during cognitive restructuring, as well as the MPFC demonstrating a reciprocal excitatory effect on the MD. Together, our findings endorse the MD as having a central role in mediating higher-order cognition by integrating and sustaining activity across widespread frontal regions. This study also provides important knowledge into the mechanistic changes that neural networks may undergo during psychotherapeutic interventions like cognitive therapy.

Neuroimaging research has provided key insights into the neural systems underpinning the regulation of affective and self-referential processing [16]. Based upon previous evidence demonstrating that the DMN is reliably activated when reflecting on one’s own traits and autobiographical knowledge [13, 14, 36, 37], we correctly hypothesized that the cognitive reframing of negative self-beliefs would elicit activity in anterior DMN regions, including the MPFC. It has been posited that the DMN contributes to the representation of the self as object, with MPFC subregions acting to select and gate these representations into conscious awareness [15]. By bringing conceptual and associative knowledge to bear on current thought and perception, this processing stream is ideally positioned to oversee introspective processes and to regulate self-referential thoughts [38]. Restructuring negative self-beliefs was also found to result in broad activation of lateral PFC control regions and anterior midline regions, namely the preSMA and dACC, which is broadly consistent with prior studies of cognitive emotion regulation [8, 10, 12]. Instead of observing suppression of DMN function, which is frequent in cognitively demanding tasks [39], restructuring negative self-beliefs evoked its joint engagement with valuation and regulatory control networks. As a whole, these effects support this novel paradigm recruiting a unique self-directed cognition whose synergistic interactions facilitate the regulation of self-representations in a constructive manner in order to enable the restructuring of negative self-beliefs.

We identified heightened activity during cognitive restructuring in subcortical structures, namely the MD, that have recently been found to form part of a more comprehensive neuroanatomical model of the DMN [40]. Extensive research from the past two decades has established a coordinating role for the MD in distinct cognitive domains that are mediated by PFC regions [19, 20, 26]. However, it has been unclear to what degree do parallel fronto-striato-thalamic circuits underlie higher-order cognitive functions, such as processing reflective cortical representations of the self. Our findings demonstrating strong excitatory effects from the MD on the MPFC, dlPFC and preSMA during cognitive restructuring directly supports recent animal work showing that the MD amplifies and sustains local PFC connectivity to enable neural sequences to emerge that maintain mental representations [41]. We posit that, rather than solely serving as a simple relay for PFC regions, excitatory MD pathways acts to increase PFC information convergence by recruiting previously untuned cortical neurons and increasing interregional synchrony, thus contributing to the generation of complex mental representations [20].

Although interactions between the thalamus and cortex are essential for cognition, there is increasing evidence that the MD may also have a role in sustaining and transmitting information on context-relevant representations to subcortical regions [26]. Our data was best explained by a model in which the MD had an excitatory effect on the caudate during cognitive restructuring, indicating that the MD may transmit updated information to the basal ganglia in support of flexible goal-directed actions. Other animal studies have demonstrated that the inhibition of MD-striatal pathways prevents the incorporation information on internal states to guide decision-making [42]. Within the context of higher-order metacognition, the MD may therefore serve as a principal integrative nexus within fronto-striatal-thalamic loops, which acts to sustain and update the value assigned to mental representations. Our model supports such an update function, whereby the MD may receive a priori predictions as input from the cortex and subsequently projects a posteriori outcomes to other regions, including those in the basal ganglia [20, 43].

MD neurons are uniquely positioned in that they are capable of representing aggregations of cortical signals [44]. Varying levels of input convergence from the PFC, both in terms of magnitude and type, endow MD circuits with a computational role that is capable of simultaneously transforming multiple modalities of information [17, 45]. Findings from our DCM analysis endorse the ventral MPFC as the primary conduit for prefrontal input onto the MD during higher-order cognition. This result aligns with recent in-vitro electrophysiological research identifying distinct roles of prefrontal-MD pathways in shaping behavior, with inhibition of ventral MPFC-to-MD pathways impacting the encoding and maintenance of contingencies [46]. The fact that the modulation of ventral MPFC-to-MD effective connectivity during cognitive restructuring was able to reliably predict participants’ tendency to engage in repetitive negative thinking suggests that this pathway may contribute to sustaining mental self-representations and, when having exaggerated impact, to rumination. That is, individuals who excessively recruit this pathway may be less disposed to cognitively reframing maladaptive negative self-beliefs and more prone to engaging in repetitive negative thinking or rumination. An alternative explanation is that this pathway reflects individual differences in the perceived difficulty of challenging negative beliefs [39, 47], as we did observe an interaction between the choice to challenge or repeat beliefs that were more strongly endorsed. Given the fundamental role of negative self-beliefs in multiple forms of psychopathology [4], these factors should be explored in future longitudinal studies assessing the malleability of neural networks underlying self-beliefs in clinical populations. Moreover, it would be of interest to determine whether the pattern of the MD having an excitatory influence on cortical activity extends beyond the regions selected in this study. Mapping the effective connectivity of additional regions using different model space configurations or other causal search algorithms (e.g., GIMME) [48], which are capable of supporting a greater number of regions than DCM, has the potential to meaningfully expand our understanding of the system of dynamic pathways mediating cognitive restructuring.

## Conclusion

Our ultra-high field fMRI study demonstrates that cognitively restructuring negative self-beliefs is supported by a distinct fronto-striato-thalamic circuitry, consistent with recent models of the self as a complex dynamic entity that emerges from the coordinated activity of multiple interacting brain systems [13–16]. Within this unique circuitry, we have confirmed a key excitatory role for the MD in humans [49], in which MD amplification acts to increase activity in multiple cortical regions during higher-order processes, as well as for the MPFC having a reciprocal excitatory effect on MD activity. Moreover, our observed relationship between MPFC-MD connectivity and individual differences in repetitive negative thinking suggests that MD pathways may represent a potential focal stimulation target for common mental health disorders [50]. Taken together, these findings advance a multifaceted framework for the MD in which it acts to increase synchrony between cortical regions to enable the generation of complex self-representations required for cognitive restructuring.

## Supplementary information

Supplementary Information

## Data Availability

The code for the effective connectivity analyses is available within the SPM12 software package (https://www.fil.ion.ucl.ac.uk/spm). Effective connectivity data and the code used to generate the results presented here are available at https://github.com/trevorcsteward/MD_Effective_Connectivity.
