# Supplementary material for: A thalamo-centric neural signature for restructuring negative self-beliefs
Source: Mol Psychiatry. 2022 Jan 1;27(3):1611–7. doi: 10.1038/s41380-021-01402-9 (PMC9095461; doi:10.1038/s41380-021-01402-9)
Supplement: Supplementary file 1 — Supplementary Information [file 41380_2021_1402_MOESM1_ESM.pdf]

Supplementary information

A thalamo-centric neural signature for restructuring negative self-beliefs

Trevor Steward[a,b], Po-Han Kung[a,b], Chris Davey[a], Bradford A. Moffat[c], Rebecca K. Glarin[c], Alec J. Jamieson[a], Kim L. Felmingham[b], Ben J. Harrison[a]

[a]Melbourne Neuropsychiatry Centre, Department of Psychiatry, The University of Melbourne & Melbourne Health, Victoria, Australia
[b]Melbourne School of Psychological Sciences, Faculty of Medicine, Dentistry and Health Sciences, University of Melbourne, Parkville, Victoria, Australia
[c]The Melbourne Brain Centre Imaging Unit, Department of Medicine and Radiology, The University of Melbourne

MRI Paradigm

Before scanning, participants were given detailed training on cognitive restructuring skills using Socratic questioning techniques, such as active rebuttal, reinterpretation, and perspective shifting [1]. After being shown a negative self-belief statement, participants were instructed to recall previous instances when the statement was not true or to imagine themselves as lawyers who must argue against the statement using factual information. To confirm participants' ability to carry out cognitive restructuring strategies, participants were asked to verbalize how they intended to mentally reframe example negative statements during scanning. Training was completed by a Masters-level clinical psychology trainee.

Prior to and following scanning, participants completed a 7-point self-rated questionnaire including the sixteen negative statements presented during the cognitive-restructuring task. Participants were instructed to indicate their level of agreement (1 = strongly disagree, 7 = strongly agree) with each statement. These statements were adapted from common negative self-beliefs described in CBT literature [1, 2], including statements such as: "I sometimes feel incompetent in the things I do" and "I think that I'm a failure". To verify the effectiveness of cognitive restructuring during the task, participant endorsement levels of negative self-beliefs pre- and post-scanning were compared using a paired sample, two-tailed, t-test.

In order to determine whether participants differed between which trials they chose to restructure or repeat beliefs, a related sample's Cochran's Q test was performed. This test determined that there was a statistically significant difference in whether participants chose to restructure or repeat a self-belief statement according to trial: $\chi2(15) = 69.558$, $p < 0.05$. Post hoc tests revealed that participants were more likely to repeat statements #2 ("I don't measure up to others.") and #11 ("People want to take advantage of me.) than to challenge them. However, after adjusting significance values by the Bonferroni correction for multiple tests (16 trials), this difference was no longer significant ($p = 0.056$).

Image Acquisition

Imaging was performed on a 7-Tesla (7T) research scanner (Siemens Healthcare, Erlangen, Germany) equipped with a 32-channel head-coil (Nova Medical Inc., Wilmington MA, USA). The functional sequence consisted of a multi-band (factor = 6) and grappa (2 times) accelerated GE-EPI sequence in the steady state (repetition time, 800ms; echo time, 22.2 ms; and pulse/flip angle, 45°) in a 20.8 cm field-of-view, with a 130 x 130-pixel matrix and a slice thickness of 1.6 mm (no gap; [3]. Eighty-four interleaved slices were acquired parallel to the anterior–posterior commissure line. A denoised T1-weighted high-resolution anatomical image (MP2RAGE; [3] was acquired for each participant to assist with functional time series co-registration (224 contiguous sagittal slices; repetition time = 5

seconds, echo time = 3.06 ms, flip angle=13°; in a 24 cm field of view, with a 256 x 256–pixel matrix and a slice thickness of 0.73 mm). To assist with head immobility, foam-padding inserts were placed either side of the participants' head. Cardiac and respiratory recordings were sampled at 50 Hz using a pulse-oximeter and respiratory belt. Information derived from these recordings were used for physiological noise correction (see below). Previous research has shown gains at 7T in statistical strength, the detection of smaller effects and group-level power using sample sizes smaller than the sample featured in the study at hand [4].

Image Preprocessing and Physiological Noise Correction

Imaging data was pre-processed using Statistical Parametric Mapping (SPM) 12 (v7771, Welcome Trust Centre for Neuroimaging, London, UK) within a MATLAB 2019b environment (The MathWorks Inc., Natick, MA). Motion artifacts were corrected by realigning each participant's time series to the mean image, and all images were resampled using 4th Degree B-Spline interpolation. Individualized motion regressors were created using the Motion Fingerprint approach to account for movement [5]. One participant was excluded due to a mean total scan-to-scan displacement over 1.6 mm (i.e., the size of one voxel). Each participant's anatomical images were co-registered to their respective mean functional image, segmented and normalized to the International Consortium of Brain Mapping template using the unified segmentation plus DARTEL approach. Smoothing was applied with a 3.2 mm^3 full-width-at-half-maximum (FWHM) gaussian kernel to preserve spatial specificity.

Physiological noise was modelled at the first level using the PhysIO Toolbox [6]. This toolbox applies noise correction to fMRI sequences using physiological recordings and has been found to improve blood-oxygen level dependent (BOLD) signal sensitivity and temporal rignal-to-noise ratio (tSNR) at 7T [7]. The Retrospective Image-based Correction function (RETROICOR; [8] was applied to model the periodic effects of heartbeat and breathing on BOLD signals, using acquired cardiac/respiratory phase information. The Respiratory Response Function (RRF; [9] convolved with respiration volume per time (RVT) was used to model low frequency signal fluctuations, which arose from changes in breathing depth and rate. Heart rate variability (HRV) was convolved with a predefined Cardiac Response Function (CRF; [10] to account for BOLD variances due to heartrate-dependent changes in blood oxygenation. Individualized DARTEL tissue maps segmented from each participant's respective anatomical scan were used to apply aCompCor, which models negative BOLD signals using principal components derived from white matter and CSF [11]. F-maps displaying group level gains following physiological noise modeling are available in Supplementary Information Fig. S1.

Dynamic Causal Modelling (DCM)

VOIs were calculated using the first eigenvariate of all voxels showing significant activation ($p < 0.05$, uncorrected) within a sphere with a radius of 4 mm around the subject specific maxima, with maxima required to be no more than 8 mm from the group maxima. To ensure that voxels included in MD VOIs did not capture adjacent regions and in accordance with recent research examining thalamic connectivity [12], time series extraction from the MD seed was run after applying an inclusive MD mask (excluding any non-MD voxels). The 1x1x1 mm^3 resolution atlas featured in the MNI-standardized Automated Anatomical Labelling 3 (AAL3) toolbox [13] was used to classify the location of the MD and only voxels falling within the medial magnocellular and lateral parvocellular nuclei of the MD were included in each VOI. Participant timeseries were pre-whitened in order to reduce serial correlations, high-pass filtered, and nuisance effects not covered by the 'effects of interest' F-contrast were regressed from the timeseries (i.e., 'adjusted' to the F-contrast).

Table S1. Descriptive Statistics

	Mean (%)	SD	Minimum	Maximum
**Sex (%)**	42.90% Female			
**Race/ethnicity (%)**	54.8% Caucasian			
	40.5% Asian			
	4.8% Other			
**Age (years)**	24.67	4.65	18	36
**Years of education**	16.56	2.64	13	23
**PTQ Total**	25.31	8.14	4	43

n=42; PTQ: Perseverative Thinking Questionnaire.

Table S2. Cognitive Reframing Task General Linear Model (GLM) Results during Chal>Rep

Region	x	y	z	T	Ke
Supp_Motor_Area_L	-8	13	69	7.85	5224
Caudate_L	-18	0	18	7.42	2924
Cerebelum_Crus1_R	42	-59	-34	6.67	2530
Temporal_Pole_Sup_L	-51	14	-21	6.18	4800
Cerebelum_9_R	2	-50	-43	5.92	313
Cerebelum_8_R	34	-64	-54	5.58	348
Temporal_Pole_Sup_R	51	11	-22	5.51	205
SN_pc_L	-6	-26	-16	5.4	61
Frontal_Mid_2_L	-37	5	61	5.35	1817
Olfactory_L	-5	22	-11	5.17	860
Caudate_R	19	-3	16	5.14	518
Cerebelum_6_L	-29	-48	-34	5.13	170
Vermis_6	5	-59	-24	4.7	639
Caudate_R	19	-24	21	4.69	51
Frontal_Mid_2_L	-29	51	6	4.67	278
Cerebelum_8_L	-30	-56	-50	4.66	93
Vermis_9	2	-53	-30	4.5	50
OFCpost_L	-32	27	-16	4.48	43
Cingulate_Mid_L	-3	-14	37	4.43	60
Cerebelum_Crus1_L	-42	-59	-26	4.41	48
Cerebelum_9_R	5	-35	-53	4.23	48
ACC_sup_R	13	18	26	4.2	72
Angular_L	-46	-64	29	4.19	266
ParaHippocampal_L	-21	-22	-27	4.17	37
Cerebelum_8_R	40	-50	-50	4.14	56
Temporal_Mid_L	-54	-56	21	4.1	38
Temporal_Sup_R	45	-32	-3	3.95	40
Frontal_Med_Orb_R	11	46	-6	3.87	60

Insula_R	37	13	5	3.85	46
Temporal_Pole_Mid_L	-34	21	-34	3.84	51
Vermis_4_5	2	-64	-10	3.61	32

All results set at a P_{FDR} < .05 threshold. Regions defined using the Automated Anatomical Labelling Atlas 3 (AAL3; local maxima labelling, 1 mm voxel edge; Rolls et al., 2020). Coordinates in MNI space.

Figure S1. Effects of PhysIO Toolbox noise correction on group-level effects

Cardiac Regressors

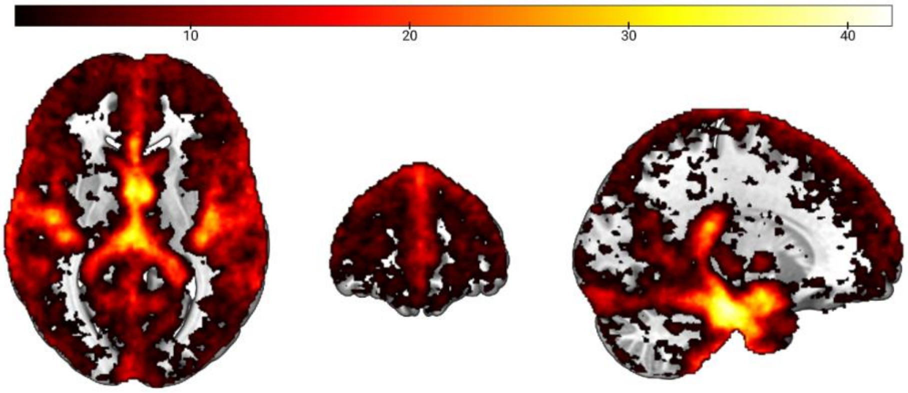

Respiratory Regressors

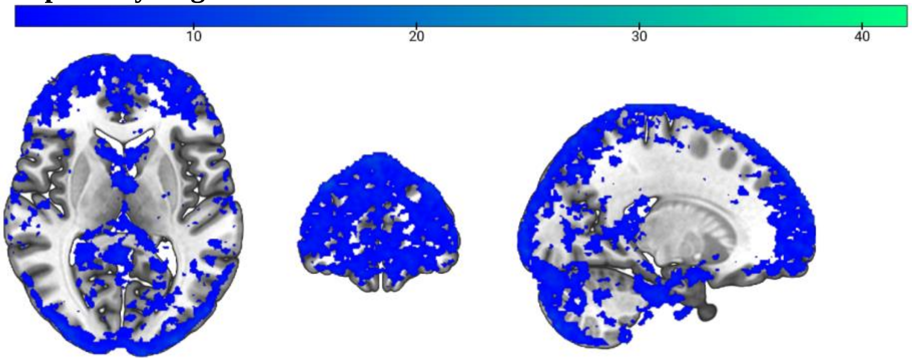

Interaction Regressors (cardiac × respiration)

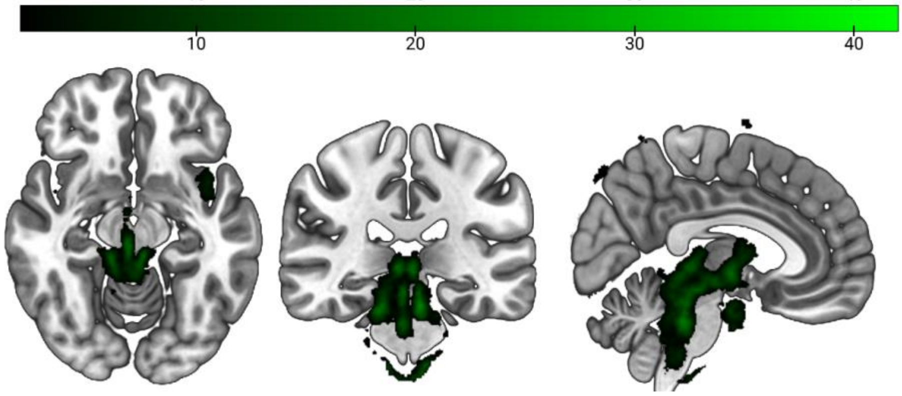

Subject count of significant physiological noise correction using the PhysIO Toolbox (Kasper et al., 2017) for each voxel in individual 1st level analyses (peak level FWE-corrected p<0.05). The top row depicts the effects of cardiac regressors, the middle row depicts the effects of respiratory regressors, and the bottom row depicts the effects of interaction regressors (cardiac × respiration). The most prominent areas (brainstem, insula, thalamus) match physiological noise sites reported in the literature (Brooks et al., 2013, Hutton et al., 2011). Color bars represent voxel-wise subject-count.

Supplementary S2. Extrinsic between-region effective connectivity and its modulatory parameters during cognitive restructuring.

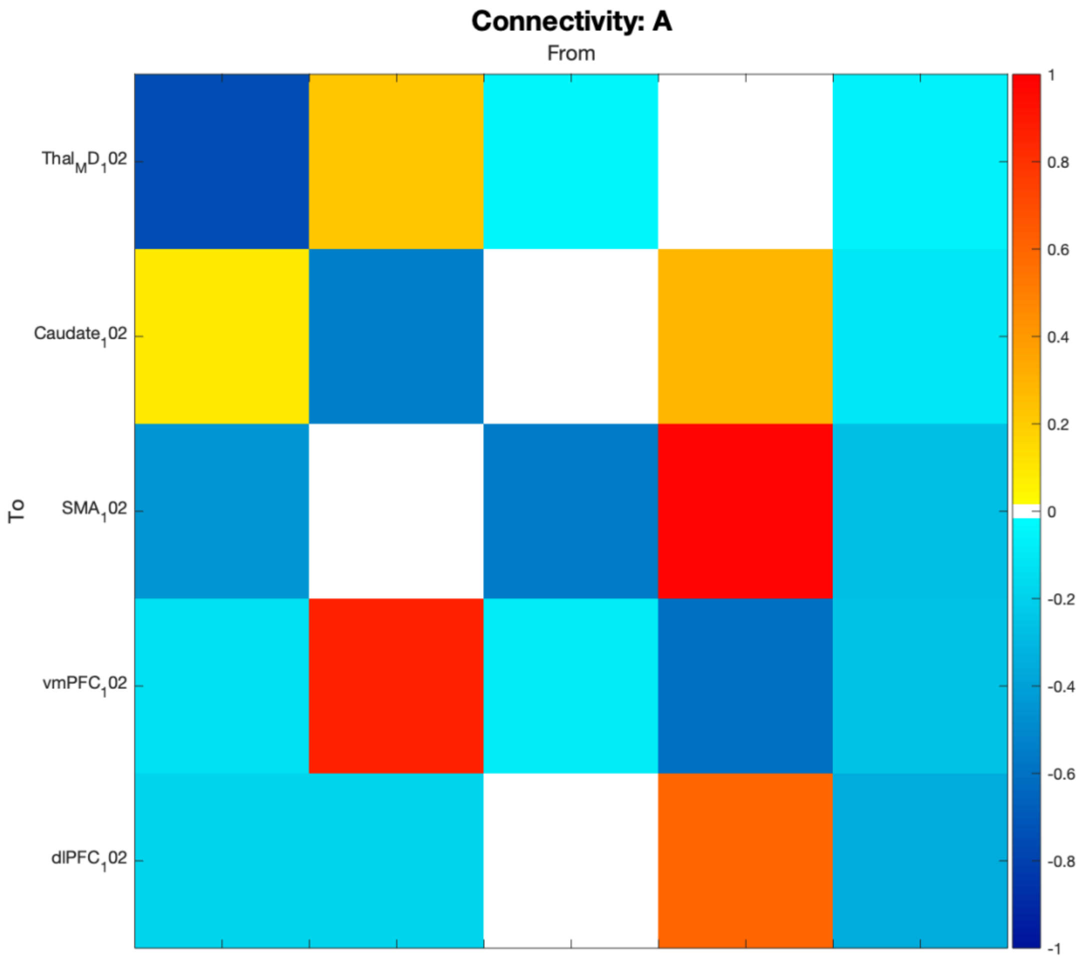

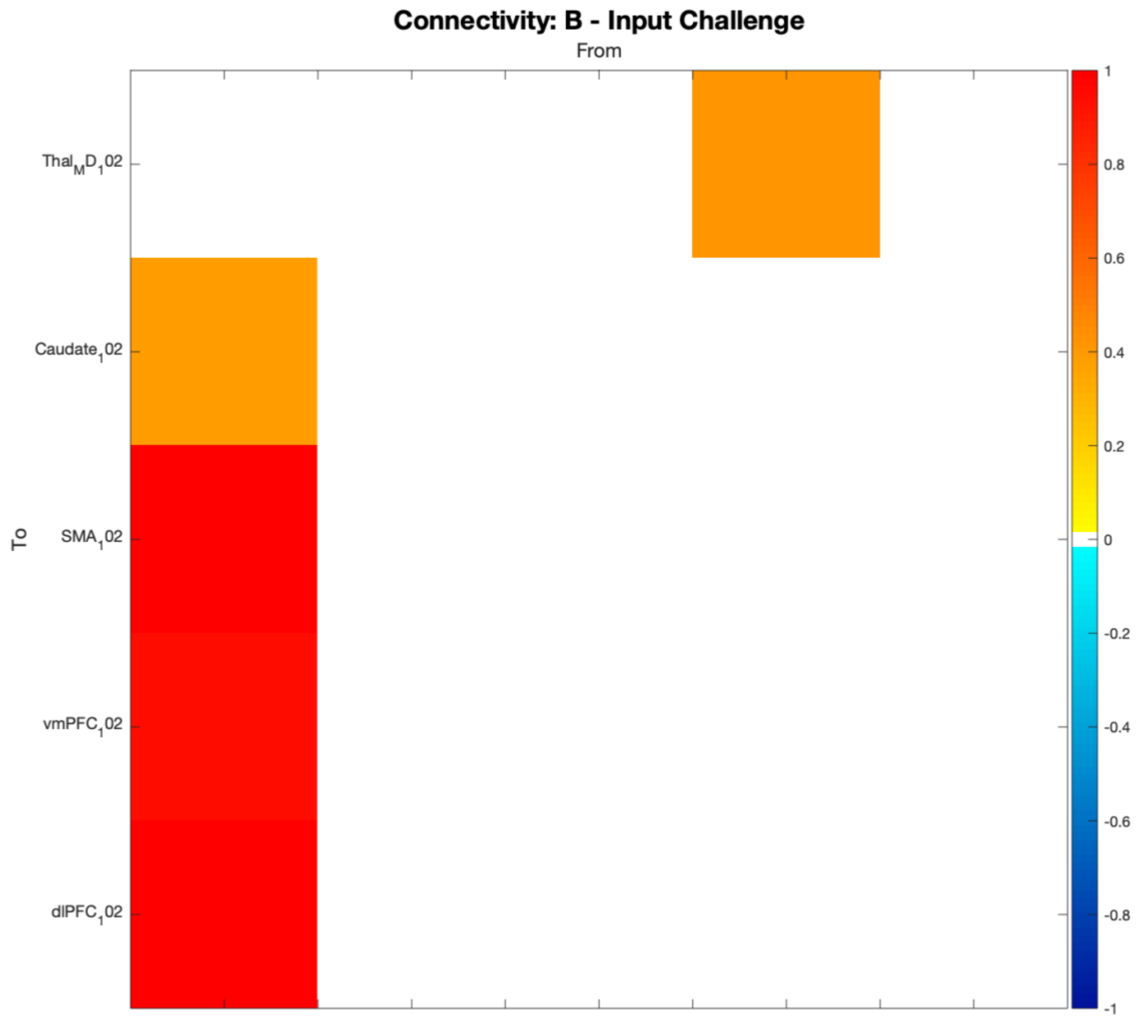

Bayesian Model Averaging (BMA) of the PEB group-level effective connectivity models afforded the 'A' parameters representing baseline effective connectivity. BMA also yielded the modulatory 'B' parameters during cognitive restructuring. The units of these effective connectivity and modulatory strengths are reported in Hertz. This figure only includes parameters related to connectivity with a posterior probability at or above 95 %. MD = mediodorsal thalamus; preSMA = presupplementary motor area; vmPFC = ventromedial prefrontal cortex; dlPFC = dorsolateral prefrontal cortex.

Supplementary S3. Modulatory parameters between the caudate, preSMA, vmPFC and dlPFC during cognitive restructuring.

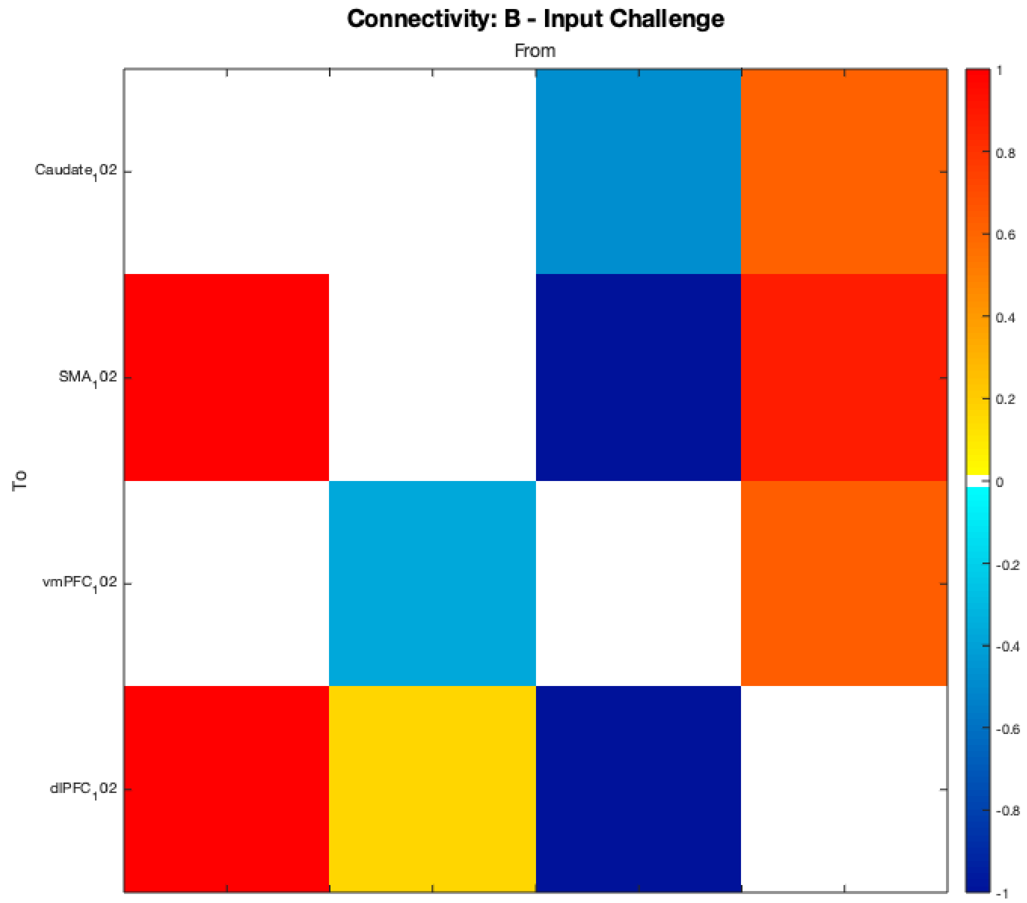

Bayesian Model Averaging (BMA) of the PEB group-level effective connectivity models afforded the modulatory 'B' parameters during cognitive restructuring. The units of these effective connectivity and modulatory strengths are reported in Hertz. This figure only includes parameters related to connectivity with a posterior probability at or above 95 %. preSMA = presupplementary motor area; vmPFC = ventromedial prefrontal cortex; dlPFC = dorsolateral prefrontal cortex.

We found evidence to support extensive modulation of cortical pathways during cognitive restructuring, with the dlPFC and the vmPFC having distinguishable excitatory and inhibitory modulatory effects on the other regions in our model space, respectively. In contrast, the preSMA had a suppressive effect on the vmPFC, while having an excitatory influence on vmPFC activity. These results align with previous DCM studies examining the modulatory effects of cognitive reappraisal, though these studies did not examine connectivity in the basal ganglia or in the MD:

Morawetz, C, et al. "Changes in effective connectivity between dorsal and ventral prefrontal regions moderate emotion regulation." Cerebral Cortex 26.5 (2016): 1923-1937.

Steward, T, et al. "Dynamic Neural Interactions Supporting the Cognitive Reappraisal of Emotion." Cerebral Cortex 31.2 (2021): 961-973.

References

1. Beck AT, Dozois DJA. Cognitive therapy: Current status and future directions. Annual Review of Medicine. 2011;62.
2. Osmo F, Duran V, Wenzel A, de Oliveira IR, Nepomuceno S, Madeira M, et al. The negative core beliefs inventory: Development and psychometric properties. Journal of Cognitive Psychotherapy. 2018;32.
3. Setsompop K, Gagoski BA, Polimeni JR, Witzel T, Wedeen VJ, Wald LL. Blipped-controlled aliasing in parallel imaging for simultaneous multislice echo planar imaging with reduced g-factor penalty. Magnetic Resonance in Medicine. 2012;67.
4. Torrisi S, Chen G, Glen D, Bandettini PA, Baker CI, Reynolds R, et al. Statistical power comparisons at 3T and 7T with a GO / NOGO task. NeuroImage. 2018;175.
5. Wilke M. An alternative approach towards assessing and accounting for individual motion in fMRI timeseries. NeuroImage. 2012;59.
6. Kasper L, Bollmann S, Diaconescu AO, Hutton C, Heinzle J, Iglesias S, et al. The PhysIO Toolbox for Modeling Physiological Noise in fMRI Data. Journal of Neuroscience Methods. 2017;276.
7. Reynaud O, Jorge J, Gruetter R, Marques JP, van der Zwaag W. Influence of physiological noise on accelerated 2D and 3D resting state functional MRI data at 7 T. Magnetic Resonance in Medicine. 2017;78.
8. Glover GH, Li TQ, Ress D. Image-based method for retrospective correction of physiological motion effects in fMRI: RETROICOR. Magnetic Resonance in Medicine. 2000;44.
9. Birn RM, Smith MA, Jones TB, Bandettini PA. The respiration response function: The temporal dynamics of fMRI signal fluctuations related to changes in respiration. NeuroImage. 2008;40.
10. Chang C, Cunningham JP, Glover GH. Influence of heart rate on the BOLD signal: The cardiac response function. NeuroImage. 2009;44.
11. Behzadi Y, Restom K, Liau J, Liu TT. A component based noise correction method (CompCor) for BOLD and perfusion based fMRI. NeuroImage. 2007;37.
12. Kafkas A, Mayes AR, Montaldi D. Thalamic-Medial Temporal Lobe Connectivity Underpins Familiarity Memory. Cerebral Cortex. 2020. 2020. https://doi.org/10.1093/cercor/bhz345.
13. Rolls ET, Huang CC, Lin CP, Feng J, Joliot M. Automated anatomical labelling atlas 3. NeuroImage. 2020;206.
